# On-chip excitation of single germanium vacancies in nanodiamonds embedded in plasmonic waveguides

**DOI:** 10.1038/s41377-018-0062-5

**Published:** 2018-09-12

**Authors:** Hamidreza Siampour, Shailesh Kumar, Valery A. Davydov, Liudmila F. Kulikova, Viatcheslav N. Agafonov, Sergey I. Bozhevolnyi

**Affiliations:** 1 Centre for Nano Optics, University of Southern Denmark, Campusvej 55, Odense M, DK-5230 Denmark; 2 L.F. Vereshchagin Institute for High Pressure Physics, Russian Academy of Sciences, Troitsk, Moscow, 142190 Russia; 3 GREMAN, Université de Tours, UMR CNRS CEA 6157, 37200 Tours, France

## Abstract

Monolithic integration of quantum emitters in nanoscale plasmonic circuitry requires low-loss plasmonic configurations capable of confining light well below the diffraction limit. We demonstrated on-chip remote excitation of nanodiamond-embedded single quantum emitters by plasmonic modes of dielectric ridges atop colloidal silver crystals. The nanodiamonds were produced to incorporate single germanium-vacancy (GeV) centres, providing bright, spectrally narrow and stable single-photon sources suitable for highly integrated circuits. Using electron-beam lithography with hydrogen silsesquioxane (HSQ) resist, dielectric-loaded surface plasmon polariton waveguides (DLSPPWs) were fabricated on single crystalline silver plates to contain those of deposited nanodiamonds that are found to feature appropriate single GeV centres. The low-loss plasmonic configuration enabled the 532-nm pump laser light to propagate on-chip in the DLSPPW and reach to an embedded nanodiamond where a single GeV centre was incorporated. The remote GeV emitter was thereby excited and coupled to spatially confined DLSPPW modes with an outstanding figure-of-merit of 180 due to a ~six-fold Purcell enhancement, ~56% coupling efficiency and ~33 μm transmission length, thereby opening new avenues for the implementation of nanoscale functional quantum devices.

## Quantum emitters: plasmonic connections

The field of integrated quantum plasmonics has taken a step forward with the demonstration of on-chip coupling between a single photon source and a plasmonic waveguide. In the approach, a nanodiamond featuring a germanium vacancy (GeV) centre that emits single photons is embedded inside a plasmonic waveguide composed of a ridge of the dielectric hydrogen silsesquioxane atop a layer of silver. Green (532 nm) laser light is coupled to one end of the waveguide via a grating and propagates to the nanodiamond where it excites the GeV centre which emits a single photon that couples into the plasmon mode of the waveguide. The researchers from Denmark, Russia and France behind the work say that the long waveguide transmission lengths (33 µm) and efficient coupling (56%) achieved open new avenues for the development of chip-based quantum circuitry.

## Introduction

Waveguides based on surface plasmon polariton (SPP) modes with inherent subwavelength confinement are fundamentally better than dielectric-based (and therefore diffraction-limited) photonic waveguides with respect to the available Purcell enhancement of the spontaneous emission rate from embedded quantum emitters^1,2^ and the current development trend towards integration and miniaturization^3–12^. Different metal-dielectric configurations have been developed that support propagating plasmonic modes confined beyond the diffraction limit, allowing for strong light–matter interaction down to the single-photon level^1,4,13–15^. Various types of SPP-based structures, such as metal nanowires (NW)^16–19^, parallel NWs^20^, V-grooves (VGs)^21^ and wedge waveguides^22^, have been demonstrated to guide single plasmons, quanta of propagating plasmonic modes, for potential quantum applications. However, the practical realization of SPP-based integrated quantum photonics has remained elusive due to several formidable challenges, including high propagation losses of SPP modes and the rather limited control over interfacing with single quantum emitters. Recently, by using relatively low-loss dielectric-loaded SPP waveguides (DLSPPWs) structured on a silver (Ag) film, simple quantum plasmonic circuits composed of embedded nanodiamonds with nitrogen-vacancy (NV) centres have been demonstrated^23,24^. The nanodiamonds hosting colour centres with their photostable single photon emission and optically readable spin states are promising candidates to build integrated quantum devices, for example, integration into plasmonic circuits. In addition to NV centres, a family of diamond colour centres based on group-IV elements in the periodic table, i.e. silicon-vacancy (SiV)^25–31^, germanium-vacancy (GeV)^32–36^ and tin-vacancy (SnV) centres^37,38^, have attracted attention due to their structural symmetries, leading to high emission into zero-phonon lines (ZPLs) accompanied by bright, spectrally narrow emission lines and indistinguishability of photons emitted from different emitters. The SiV centres exhibit an optical transition at a longer wavelength (ZPL at 738 nm) operating with smaller SPP loss in the metal^39^. At the same time, the SiV excited state decay is dominated by the nonradiative relaxation, causing lower quantum efficiency for SiV centres^40^. More recently, the GeV centres in diamond have shown higher quantum efficiency and a larger absorption cross section than SiV centres^33^. Having such single-photon emitters in diamond nanocrystals becomes very important when it comes to the implementation of hybrid quantum-plasmonic systems and can facilitate remote excitation of GeV centres incorporated in a plasmonic chip.

**Fig. 1:**
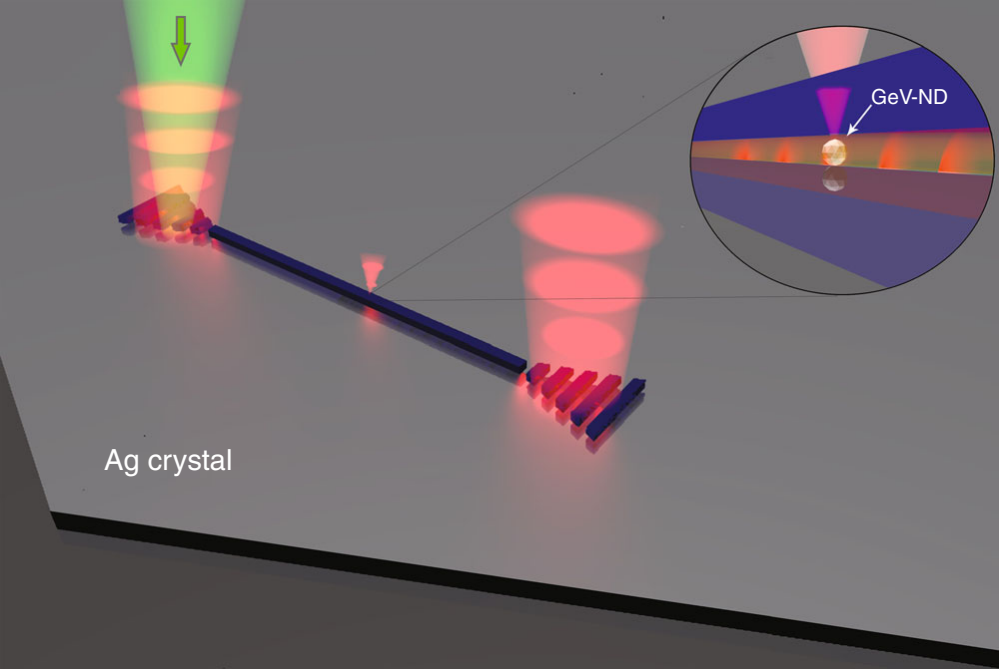
Schematic of the device layout and working principle for on-chip excitation of a nanodiamond (ND) carrying spectrally narrow single GeV quantum emitters embedded in a DLSPP waveguide. A 532-nm pump laser light is coupled with a grating, propagates on-chip in the low loss DLSPPW and reaches an embedded nanodiamond that contains a single GeV centre (GeV-ND). The remote GeV emitter is thereby excited, generating single optical plasmons propagating along the waveguide and outcoupling from the ends

In this work, we demonstrate on-chip generation and propagation of spectrally narrow single optical plasmons excited by GeV centres in nanodiamonds using DLSPPWs. The synthesis and detailed characterization of the GeV nanodiamonds are presented here, for the first time to our knowledge, describing specific features useful for working with these colour centres. The nanodiamonds were produced using the high-pressure high-temperature (HPHT) method, and Ge was introduced during the growth process to incorporate single GeV centres (see details in the Materials and methods section). Furthermore, we propose and demonstrate a hybrid approach using DLSPPWs structured on single Ag crystals that feature considerably lower SPP damping rates in comparison with Ag films fabricated by other techniques^41–47^. The latter allows us to realize sufficiently long SPP propagation at both the excitation and emission wavelengths of GeV centres, thereby facilitating the remote excitation of GeV centres in nanodiamonds incorporated in a plasmonic chip. The colloidal Ag crystal flakes were synthesized using a recently reported method^47^. Figure 1 shows a schematic of the device layout, introducing the working principle of on-chip remote excitation. The hybrid plasmonic configuration enabled the green laser light to propagate on-chip, in the DLSPPW, and reach to an embedded nanodiamond containing a single GeV centre. The remote GeV emitter is thereby excited, and single photons are channelled to a DLSPPW mode. Finally, the ability of GeV–DLSPPW system for efficient long-range delivery is compared with other hybrid quantum plasmonic systems, revealing an exceptional figure of merit (FOM) of 180 due to a ~six-fold Purcell enhancement, ~56% coupling efficiency and ~33 μm transmission length at *λ* = 602 nm (ZPL, GeV).

## Results

**Fig. 2:**
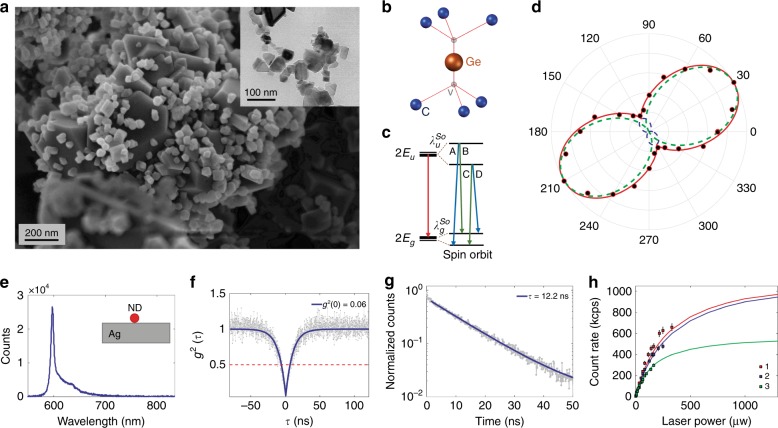
Characterization of single GeV centres in HPHT nanodiamonds. **a** Scanning electron microscopy (SEM) image of the GeV nano and microdiamonds performed on the ‘raw’ sample (after HPHT synthesis). Inset shows a transmission electron microscopy (TEM) image of the GeV nanodiamonds of different sizes taken after the chemical and ultrasonic treatment. **b** The Ge atom is located in the middle of two empty lattice sites, which includes inversion symmetry. **c** Electronic structure and optical transitions of the GeV centre. Optical transitions B and C have polarization axes parallel to the symmetry axis of the system, and transitions A and D are perpendicular to the symmetry axis, indicating that the GeV emitter has two orthogonal dipoles. **d** Normalized photon rate for a single GeV nanodiamond on the Ag plane versus analyser angle, measured (dot) and model fit (solid). Dashed curves indicate contributions of the two orthogonal dipoles. **e**–**g** Spectrum (**e**), second-order correlation (**f**) and lifetime (**g**) measurement results taken for the GeV nanodiamond. The integration time on the spectrum is 300 s, and the excitation power is 10 μW. **h** Saturation curves are taken for three different single GeV nanodiamonds on the Ag-coated substrate that are fitted to the model of *C(P*) = *C*_∞_*P*/(*P* + *P*_sat_), in which *C* is the count rate, *P* denotes the excitation power, *P*_sat_ is the saturation power and *C*_∞_ indicates the asymptotic count rate^60^. The measured data indicate ultra-bright single photon sources based on GeV centres in nanodiamonds

A scanning electron microscopy (SEM) image of the GeV nano and microdiamonds in the ‘raw’ sample after HPHT synthesis is shown in Fig. 2a. The inset shows a transmission electron microscopy (TEM) image of the GeV nanodiamonds of different sizes (from 20 to 120 nm) taken after the chemical and ultrasonic treatment. Chemical treatment was carried out with three highly concentrated acids, HNO_3_, HClO_4_ and H_2_SO_4_ (at 200 °C for 3 h), to remove traces of graphite. The ultrasonic treatment was done with a UP200H device (Hielscher). The Ge atom is located in the middle of two empty lattice sites, which includes inversion symmetry (Fig. 2b), and the system has an electronic structure and optical transitions (Fig. 2c) similar to reports for the group-IV family of diamond colour centres^29,33,37,48^.

In the first designed experiment, a Ag-coated silicon wafer was patterned with regularly placed gold markers^23,24^, and the synthesized GeV nanodiamonds were spin coated onto this substrate. A 1-nm layer of poly(allylamine hydrochloride) (PAH) was put on the Ag layer to improve the distribution and attachment of the nanodiamonds to the Ag surface^49^. The sample was then raster scanned using confocal fluorescence microscopy. Lifetime (Fig. 2g), spectrum (Fig. 2e), autocorrelation (Fig. 2f) and saturation curve (Fig. 2h) measurements were taken for the single GeV nanodiamonds on the Ag film. The results indicate ultrabright, spectrally narrow and stable single photon sources in the nanodiamonds. We also measured the polarization characteristics of GeV nanodiamonds deposited on a Ag-coated substrate, using an analyser in the detection pathway to determine the projection of polarization axes of single photons emitted on the surface plane (see the experimental setup in Supplementary Materials, Figure S1). The measured polarization data for a single GeV nanodiamond are shown in Fig. 2d and well-fitted to the model of two orthogonal dipoles, following the polarization characteristics of the group-IV colour centres^28,50^.

**Fig. 3:**
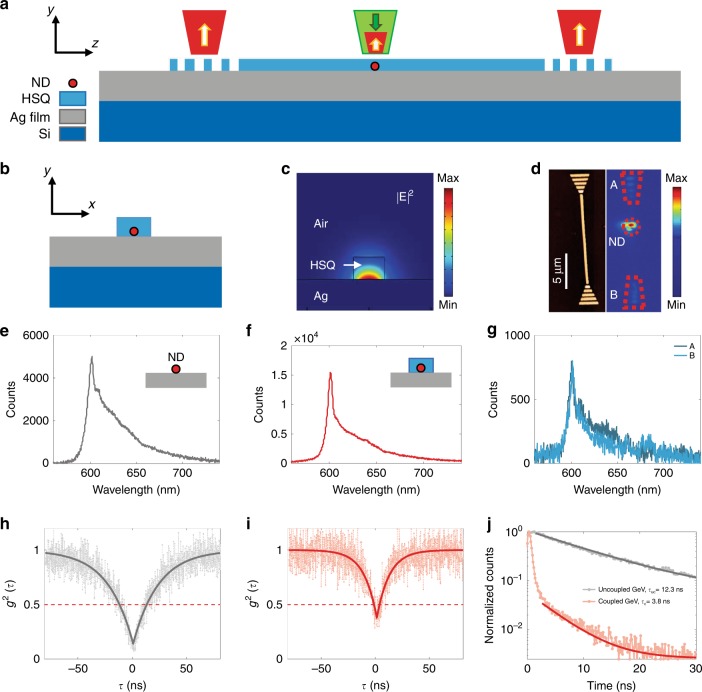
On-chip direct excitation of a single GeV nanodiamond. **a, b** Schematic of a sample layout and the working principle of direct excitation of a GeV nanodiamond embedded in a plasmonic waveguide. **c** Simulated mode profile for the DLSPP waveguide at *λ* = 602 nm (GeV ZPL). **d** AFM image of the fabricated waveguide (left), and CCD camera image of the whole structure where the nanodiamond is excited and a fluorescence image of the focal plane is taken (right). **e**–**g** Spectrum taken from uncoupled GeV (**e**), coupled GeV (**f**) and outcoupled light through the grating ends A and B (**g**). The integration time of the spectra is 300 s, and the excitation power is 25 μW. **h**, **i** Second order correlation of the GeV centre before (**h**) and after (**i**) coupling to the waveguide. **j** Lifetime of the GeV centre taken before (grey) and after (red) coupling

The position of nanodiamonds containing single GeV centres was determined with respect to the gold markers. Using electron beam lithography with hydrogen silsesquioxane (HSQ) resist^23^, waveguides are fabricated on Ag-coated substrate to contain those nanodiamonds that are found to feature appropriate single GeV centres. Controlled placement of nanodiamonds in plasmonic nanostructures is explained in the Supplementary Materials (Figure S6). Our deterministic technology provides a ~30 nm precision in placement that can further be enhanced by using the SEM image. This is limited by the size of nanodiamonds, which can be reduced down to 1 nm, achieved by current diamond synthesis technology^51,52^. A schematic of the device layout for on-chip direct excitation of nanodiamonds is illustrated in Fig. 3a, and Fig. 3b, c shows a distribution of normalized |*E*|^2^ (where *E* is the electric field) of the DLSPPW mode with a cross-section of 180 nm in height and 250 nm in width of HSQ (dielectric constant of 1.41) on the Ag surface^53^ at *λ* = 602 nm. An atomic force microscopy (AFM) image of the HSQ waveguide is presented in Fig. 3d (left). The whole structure, when the GeV centre was directly excited with green pump laser, was imaged using a charge coupled device (CCD) camera. The image shows three spots, ND, A and B (Fig. 3d, right). This shows excitation and emission of the GeV emitter (ND), coupling of GeV emission to the DLSPPW mode, propagation and out-coupled radiation from the two ends (A and B). The emission spectra of the uncoupled (Fig. 3e) and coupled GeV (Fig. 3f) indicate a ~3 times enhancement in the emission rate of the GeV centre after coupling to the confined DLSPPW mode (for the same excitation power of 25 μW and an integration time of 300 s). Figure 3g shows the spectra from the out-coupling grating couplers A and B, indicating the on-chip transmission of the spectrally narrow surface plasmon mode excited by the GeV emitter. The antibunching dip of the correlation function, before (Fig. 3h) and after (Fig. 3i) coupling, indicates a single GeV emitter (*g*^2^ (*τ* = 0) < 0.5). The data are analysed with a single exponential fitting curve^33^. The lifetime of the GeV centre before (Fig. 3j, grey) and after (Fig. 3j, red) coupling is measured, indicating a reduction (from ∼12.3 to ∼3.8 ns) for the coupled GeV centre. The lifetimes are obtained by tail fitting of the measured data with a single exponential curve^23^. The lifetime decreased by a factor of ∼3 ± 0.5, which is in addition to the two times reduction due to the Ag plane (Supplementary Materials, Figure S2), giving, on average, a ∼six-fold Purcell enhancement.

We estimated the propagation length of the DLSPPW on the Ag film using the fluorescence intensities at the two ends of the waveguide^23^. We obtain the propagation length of 9 ± 3 μm for the GeV–DLSPPW hybrid system on the Ag film, which is smaller than the NV–DLSPPW system^23^ due to the higher SPP loss at the lower wavelength region corresponding to emission from GeV centres.

**Fig. 4:**
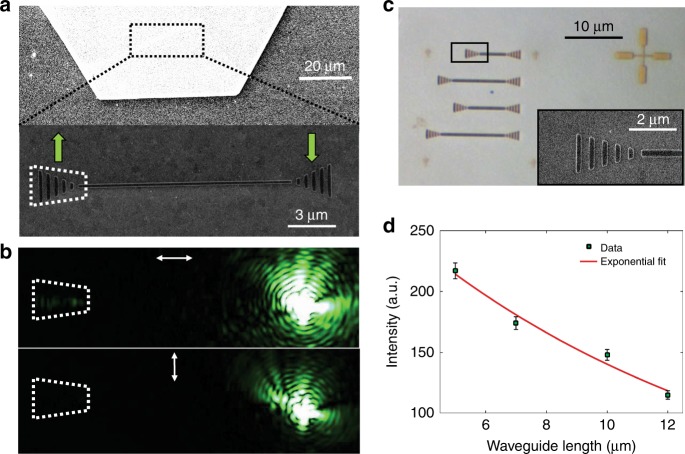
Transmission of the 532-nm pump laser light along the low-loss plasmonic waveguide. **a** SEM image of a single crystalline Ag flake (top) and fabricated DLSPP waveguide on the Ag plate (bottom). **b** Optical characterization of the waveguide for parallel (top) and perpendicular (bottom) polarizations of 532-nm laser light. **c** Bright-field microscopy image of fabricated waveguides on the Ag flake with different lengths. The inset shows an SEM image of the grating couplers at the end of the waveguides with an optimized taper opening angle of 30° that maximize the in-coupling efficiency of DLSPPWs (see details in refs. ^54,55^). **d** Measured data for propagation characteristics of the DLSPPWs on Ag crystals with different lengths (green squares) and exponential fitting curve (red line), giving the propagation length of 11.8 μm at *λ* = 532 nm (pump laser) for the DLSPPW on the Ag flake

Using a single-crystalline Ag flake instead of the Ag film significantly enhanced the DLSPPW propagation length (Fig. 4). Figure 4a shows an SEM image of a HSQ waveguide fabricated on a Ag crystal flake. Optical characterization of the waveguide shows transmission of green laser light (532 nm) through the DLSPPW mode for the polarization along the waveguide axis (Fig. 4b). We have measured the transmission for several waveguides with different lengths (Fig. 4c, d), achieving an extraordinary long propagation length of ~11.8 μm for the green laser light through the low-loss DLSPPW (see Supplementary Figure S4 for the characterization of DLSPPW without grating couplers). The funnel tapering dimensions of the grating coupler (Fig. 4c, inset) are optimized to obtain the in-coupling efficiency of ∼12% extracted from the fitting curve shown in Fig. 4d using the well-established cutback method^54,55^.

**Fig. 5:**
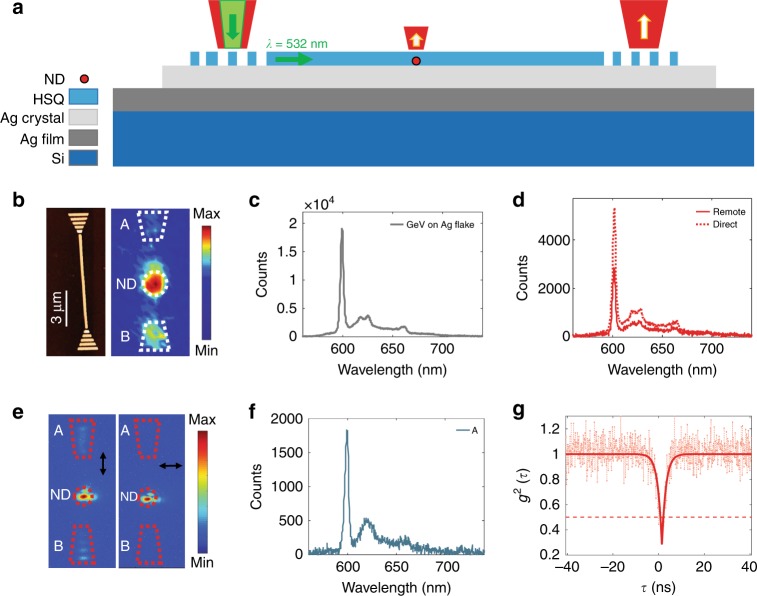
On-chip remote excitation of a single GeV nanodiamond. **a** Schematic of a sample layout for on-chip remote excitation of a GeV nanodiamond embedded in the plasmonic structure. **b** AFM image of the fabricated waveguide (**b**, left) and galvanometric mirror scan image showing the remote excitation of the embedded GeV where the pump laser light is illuminated at end B (**b**, right). Higher emission at end B is caused by the background fluorescence from the grating coupler exposed to the strong pump light. **c**, **d** Spectra taken from the uncoupled GeV, i.e. the nanodiamond on the Ag plate (**c**) and from coupled GeV when excited remotely (**d**, solid line) and in the case of direct excitation (**d**, dotted line). **e** CCD images for the coupled system when excited directly and with a linear polarizer placed in the detection path are presented for two orthogonal polarizations, parallel (left) and perpendicular (right) to the waveguide axis. **f** Spectrum taken from the outcoupled light through grating end A in the case of remote excitation. The integration time on the spectra data is 300 s, and the excitation powers are 2 μW (**c**, **d**) and 5 μW (**f**). **g** Second order correlation function of the GeV emitter confirming a single photon emission

In the second designed experiment, we employ the capability of green light transmission in the DLSPPW on Ag crystals and demonstrate remote excitation of GeV nanodiamonds embedded in low-loss DLSPPWs. We spin-coated a small amount of the synthesized solution of Ag crystal flakes on a Ag-coated silicon wafer. A PAH layer is put on the Ag film to improve the adhesion of the Ag flakes to the substrate^49^. The markers are fabricated on Ag flakes, and the nanodiamonds with GeV centres are spin-coated. The sample fluorescence is then imaged using confocal microscopy. Spectra and correlation functions are taken for the nanodiamonds on Ag crystals. Using electron beam lithography, the HSQ waveguides are fabricated on the Ag crystal, embedding the selected single GeV nanodiamonds. A schematic of the device layout and working principle for on-chip remote excitation is illustrated in Fig. 5a. Figure 5b shows an AFM image of the fabricated waveguide (left) and a galvanometric mirror scan image (right) when the waveguide is excited from end B with the green pump laser and the fluorescence emission detected at the focal plane. Emission from the embedded GeV nanodiamond located inside the waveguide confirms a remote excitation of the GeV centre, and out-coupled radiation from the two ends (A and B) indicates the coupling of the GeV centre to the DLSPPW mode. Figure 5c illustrates the spectrum of the GeV emitter before coupling (nanodiamond on the Ag crystal). The emission spectra after coupling for the remotely excited GeV centre (solid line) and the spectrum for the same GeV centre when excited directly (dotted line) is presented in Fig. 5d. CCD images for the coupled system when excited directly and with a linear polarizer placed in the detection path are presented in Fig. 5e. A comparison of the two images in Fig. 5e clearly indicates a strongly polarized emission from the end of the waveguide due to the coupling of the emission to the fundamental transverse magnetic (TM) mode of the DLSPP waveguide, propagation and subsequent scattering from the ends. Figure 5f shows the spectrum measured at the grating end A, when the GeV centre is remotely excited (Fig. 4b). In Fig. 5g, we present the second-order correlation function for the GeV centre, confirming single photon emission (*g*^*2*^(*τ* = 0) < 0.5). Apparent differences in the second-order correlation functions shown in Figs. 3i and 5g are related to a large dispersion in the fluorescence lifetimes of the GeV centres in the nanodiamonds (Supplementary Figure 2a).

**Fig. 6:**
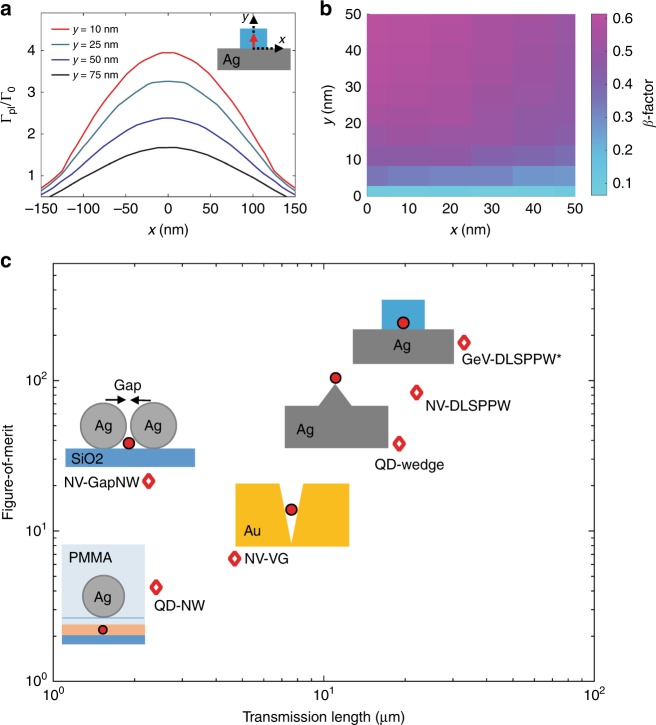
Efficiency of the GeV-DLSPPW platform compared with other hybrid quantum systems. **a** Simulated plasmonic decay rate (Γ_pl_/Γ_0_) for the DLSPPW coupled GeV centre. Inset shows the cross section of a *y*-oriented dipole emitter inside the DLSPP waveguide (top right). **b** Distribution profile of the *β*-factor, i.e. Γ_pl_/Γ_tot_, for a distribution of the GeV centre inside a nanodiamond, where each coloured square represents the central value of the corresponding in-plane dipole position. **c** Figure-of-merit (FOM) and transmission length of hybrid quantum plasmonic systems. The FOM of GeV–DLSPPW on the Ag crystal is compared with other demonstrated quantum emitter-plasmonic waveguide (QE-PW) hybrid systems, including quantum dot-Ag nanowire (QD-NW)^16^, NV-Gap Ag nanowire (NV-GapNW)^20^, NV-V groove channel waveguides (NV-VG)^21^, QD-Wedge waveguides (QD-wedge)^22^ and NV–DLSPPW on a Ag film^23^. The black diamond markers in the graph are extracted from the experimental results reported for the corresponding hybrid systems

We simulated the GeV decay rate into the DLSPPW mode using the finite-element modelling (FEM) method^23,56^. The GeV centre was modelled as a single dipole emitting at 602 nm in the simulations. The effect of the orientation of the GeV dipole axis on the decay channels is discussed in the Supplementary Materials (Figure S3). An up to four-fold decay rate to the plasmonic mode is predicted for a GeV centre in the waveguide compared to its emission in vacuum. Figure 6a shows the plasmonic decay rate dependence for the optimum orientation of the dipole on its position in the waveguide. The emission efficiency (*β* factor) of the emitter to the DLSPPW mode is given by *β* = Γ_pl_/Γ_tot_, where Γ_tot_ denotes the total decay rate, and Γ_pl_ is plasmonic decay rate^23^. The total decay rate is calculated from the time evolution of the total dissipated power^23,56,57^. The *β* factor for a *y*-oriented GeV coupled to a DLSPPW is simulated as a function of the position in the cross-section (Fig. 6b). Palik’s data^53^ are used for modelling of the Ag refractive index. The simulated results show that the *β* factor can reach 62%. We measure the apparent *β* factor (*β*_pl_) using *β*_pl_ ≃ (*I*_A_ + *I*_B_)/(*I*_A_ + *I*_B_ + *I*_GeV_), in which *I*_A_ and *I*_B_ are the out-coupled radiation at the ends A and B, respectively, and *I*_GeV_ is the measured intensity at the GeV centre position^23^. This gives a *β* factor of 56% for the GeV–DLSPPW system shown in Fig. 5, which is in good agreement with the simulated *β* factor, indicating accurate alignment of the waveguide with respect to the GeV nanodiamond. We measured the 1/*e* propagation length of the GeV emission along the DLSPPW on Ag crystal, the same way as described for Ag film, and obtained a value of 33 ± 3 μm (Supplementary S5). This is significantly larger than DLSPPW on the polycrystalline Ag film and even higher than the propagation length of NV–DLSPPW^23^, indicating low material loss for the single crystalline Ag flakes.

## Discussion

The ability of a hybrid plasmonic system to realize efficient single-photon transmission can be characterized with the figure of merit (FOM = 1/*λ β L*_P_ Γ_tot_/Γ_0_)^21,23^. The GeV–DLSPPW hybrid system reaches a FOM value of 180 ± 25 (Γ_tot_/Γ_0_ = 6 ± 1, *β* = 0.56 ± 0.03, *L*_P_ = 33 ± 3 μm and *λ* = 602 nm), clearly outperforming previous demonstrations of quantum emitter-plasmonic waveguide (QE-PW) coupled systems^16,20–23^. A careful comparison of GeV–DLSPPW on the Ag crystal with other hybrid systems of QE-PW is presented in Fig. 6c. The efficiency of the light–matter interaction in the GeV–DLSPPW platform can also be compared to other colour centre photonic platforms using the so-called cooperativity parameter (defined as Γ_w_/(Γ_tot_–Γ_w_), in which Γ_w_ is the decay rate into a waveguide mode). For the SiV-centre incorporated cavity system described in ref. ^25^ and for the GeV–based platform presented in ref. ^33^, the cooperativity of *C* = 1 and *C* = 0.1 have been deduced, respectively. The cooperativity was estimated to be *C* = 1.5 in this work using the GeV–DLSPPW platform, which should be understood as the upper cooperativity limit evaluated from our experiment at room temperature (as opposed to the cooperativity estimated at low temperatures^25,33^). This can be enhanced further by using the waveguide-integrated cavity resonator^24^ and/or using dielectric ridges with a larger refractive index (e.g. TiO_2_ with a refractive index of ~2.4) and smaller cross section DLSPPW mode (and therefore stronger coupling).

In conclusion, we have demonstrated on-chip generation and transmission of spectrally narrow single optical plasmons excited by GeV nanodiamonds embedded in DLSPPWs. The extraordinary long propagation length for the green pump laser has been achieved with DLSPPWs on Ag crystals, enabling thereby the remote excitation of GeV centres through propagating DLSPPW mode in a plasmonic chip. The performance of the GeV–DLSPPW hybrid system with respect to efficient single-photon transmission has been quantified with the FOM of 180 by a ~six-fold Purcell enhancement, ~56% coupling efficiency and ~33 μm transmission length, which indicates a superior performance in comparison with the previously demonstrated systems. Further enhancement of the Purcell factor is possible by using a larger refractive index dielectric (for example, TiO_2_ with a refractive index of ~2.4 at the wavelength of 600 nm) with smaller dimensions in the DLSPPW cross section. Our demonstration opens the way for the integration of an excitation laser, quantum emitter and plasmonic circuit on the same chip. Detection of single plasmons and two-plasmon interference have already been demonstrated on a chip^58,59^. With the combination of all these technologies, it will be possible, in the near future, to have all the elements of a quantum plasmonic circuit integrated on a chip.

## Materials and methods

### GeV nanodiamond synthesis

HPHT synthesis of nanodiamonds with GeV centres has been realized based on the hydrocarbon metal catalyst-free growth system. Tetraphenylgermanium C_24_H_20_Ge (Sigma-Aldrich) was used as the initial germanium-containing hydrocarbon compound. The synthesis was performed in a high-pressure apparatus of the Toroid type. Cylindrical samples of the initial material (5 mm in diameter and 3 mm in height) obtained by cold pressing were put into graphite containers serving as heaters for the high-pressure apparatus. The containers were loaded in the apparatus up to 8 GPa and heated up to the synthesis temperature under a constant load for 1–5 s. The obtained diamond products were then isolated and characterized with X-ray diffraction, Raman spectroscopy, SEM and TEM. The results of such characterization of the obtained products, which are mixtures of ultranano-, nano- and submicrometre-size fractions of diamond, show high, practically 100%, yield in the formation of diamond. Size-fractional separation of diamonds was carried out in several stages that consisted of ultrasonically dispersing the diamond particles using UP200Ht dispersant (Hielscher Ultrasonic Technology), chemical treatment of the samples in a mixture of three acids (HNO_3_–HClO_4_–H_2_SO_4_), and subsequent centrifugation of an aqueous or alcohol dispersion of diamond powders.

## Electronic supplementary material

Supplementary Materials
