# Supplementary material for: On-chip excitation of single germanium vacancies in nanodiamonds embedded in plasmonic waveguides
Source: Light Sci Appl. 2018 Sep 12;7:61. doi: 10.1038/s41377-018-0062-5 (PMC6134053; doi:10.1038/s41377-018-0062-5)
Supplement: Supplementary file 1 — Supplementary Materials [file 41377_2018_62_MOESM1_ESM.pdf]

Supplementary Materials for

On-chip excitation of single germanium vacancies in nanodiamonds embedded in plasmonic waveguides

Hamidreza Siampour[*,1], Shailesh Kumar[1], Valery A. Davydov[2], Liudmila F. Kulikova[2], Viatcheslav N. Agafonov[3], and Sergey I. Bozhevolnyi[1]

[1] Centre for Nano Optics, University of Southern Denmark, Campusvej 55, Odense M, DK-5230, Denmark

[2] L.F. Vereshchagin Institute for High Pressure Physics, Russian Academy of Sciences, Troitsk, Moscow, 142190, Russia

[3] GREMAN, UMR CNRS CEA 6157, Université de Tours, 37200 Tours, France

[*]E-mail: hasa@mci.sdu.dk

S1. Experimental setup

The experimental set-up used for characterization of the germanium vacancy (GeV) centers and their coupling to dielectric loaded surface plasmon polaritons waveguide (DLSPPW) is presented in Figure S1.

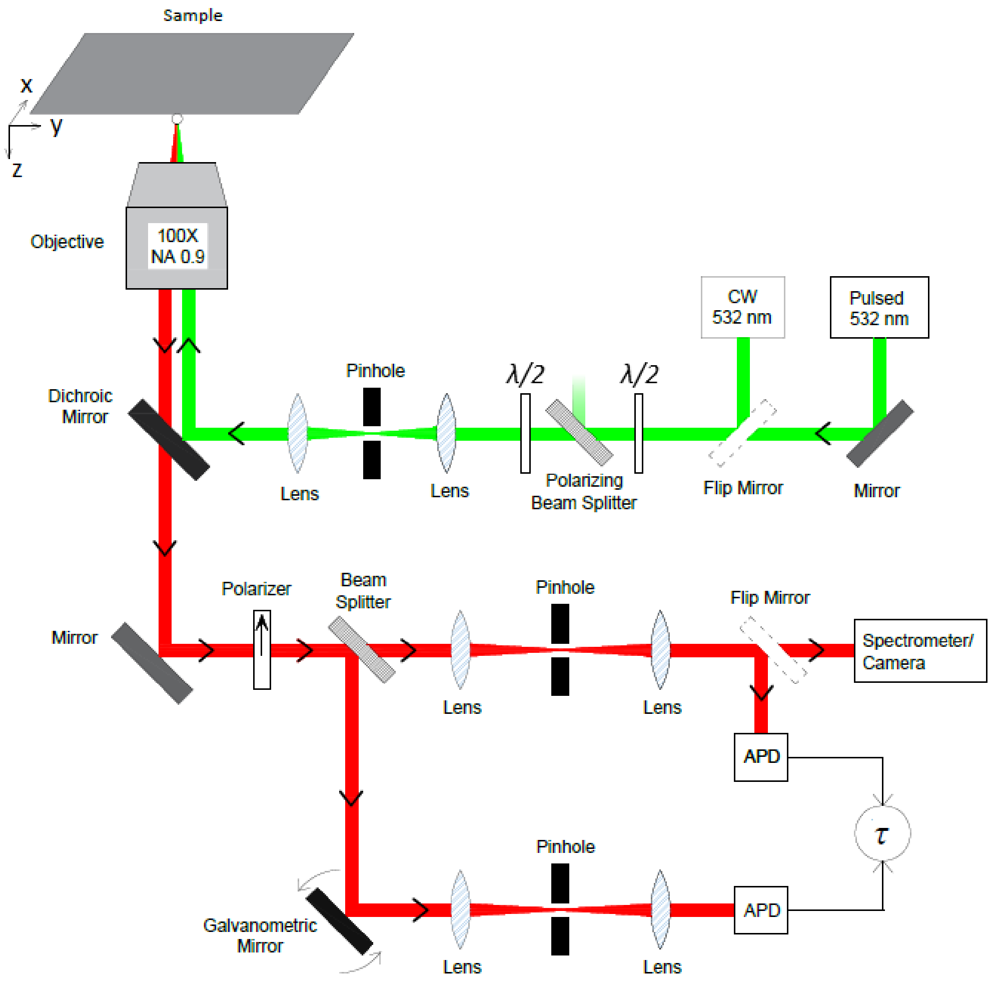

Figure S1 Schematic of experimental setup for quantum measurements. Green line indicates excitation path from 532 nm continuous-wave (CW) or pulsed lasers (chosen by a flip mirror) onto the sample, which is focused by a 100× (NA 0.90) objective. The pump polarization is controlled by a halfwave plate in the excitation light path. The fluorescence light, indicated by red line, is collected by the same objective, and passed through a dichroic mirror, polarizer (analyser) and then beam splitter. The analyzer introduced in the detection path probes the polarization of emitted photons. When illuminated by a CW laser, the emission from a single quantum emitter is split into two channels through the beam-splitter and then detected by two

identical avalanche photodiodes (APDs) where we record time delay across the APDs to generate an intensity autocorrelation signal $g^2(t) = <I(t')I(t'-t)>$. Lifetime measurements are performed using pulsed excitation with pulse width/period of ~50 ps/400 ns. Postfabrication measurements are performed to show coupling of the emitter to the DLSPP waveguide where the nanodiamond is excited and a fluorescence image of the focal plane is taken either by a charge-coupled device (CCD) camera or a galvanometric mirror scan. Fluorescence spectrum of GeV-waveguide system is taken by a grating spectrometer.

S2. Distribution of fluorescence lifetimes for GeV emitters in nanodiamonds

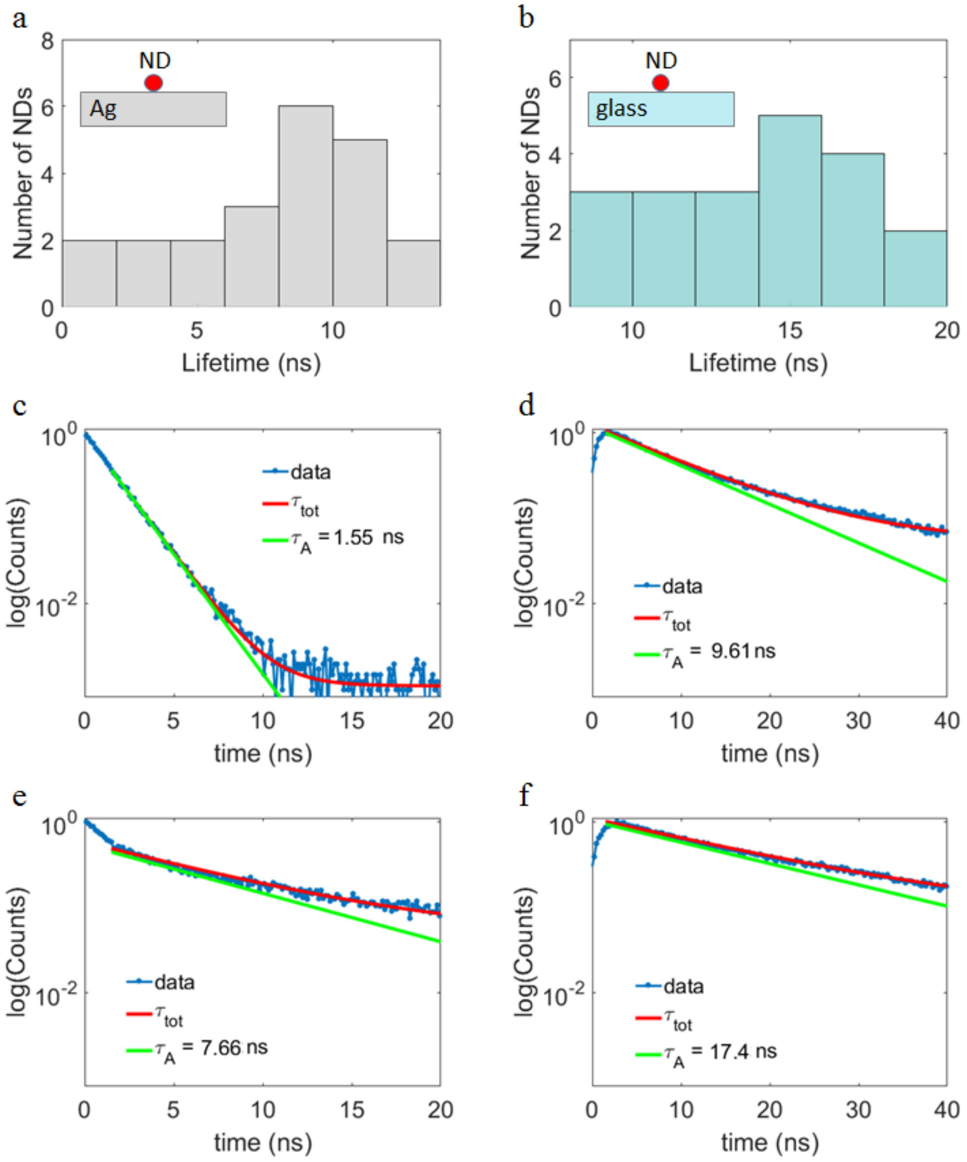

Figure **S2** (**a**, **b**) Histogram graphs for lifetime distribution of GeV nanodiamonds on Ag surface (a), and on glass (b). Average lifetime of GeV nanodiamond on Ag plate is calculated to be 8.1. We have also calculate lifetimes for bare NDs (NDs on glass), where there is no influence from Ag plate and observed longer lifetimes (~15.9 ns on average), suggesting a 2-fold lifetime reduction due to the Ag plate. (**c**, **e**) Lifetime measurements on Ag plate for two cases in short range (c) and middle range of our collected data (e). τ_{tot} denotes total decay calculated from single exponential fit ($\tau_{tot}=A.\exp(-t/\tau_A)+c$, in which c is constant). (**d**, **f**) Lifetime measurements on glass for two cases in short range (d) and middle range of our collected data (f).

S3. Effect of GeV dipole axis on plasmonic decay rate

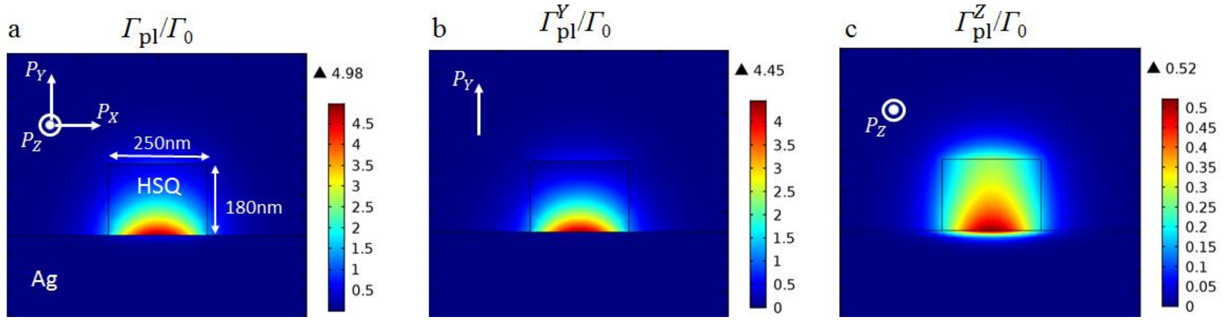

Figure **S3**: Simulated plasmonic decay of coupled GeV center for different polarization axes. (**a**-**c**) Plasmonic decay (Γ_{pl}/Γ_0) of GeV emission to the fundamental transverse magnetic (TM) mode of the DLSPP waveguide is maximized for the polarization axis normal to the Ag plane, i.e. y-axis (**b**). There is also a ~10% contribution from in-plane polarization axis along the waveguide axis (z-axis) to the DLSPPW mode (**c**) which can be added efficiently in the plasmonic decay by proper alignment of the waveguide axis along the dominant dipole component (e.g. along $\theta = \pi/6$ in the GeV nanodiamond shown in Figure 2d in the manuscript). Dipole axis has similar effect on β-fator (Γ_{pl}/Γ_0), i.e. the main contribution is belong to the normal axis (y-axis) polarization.

S4. Propagation characteristics of DLSPPW on Ag crystals

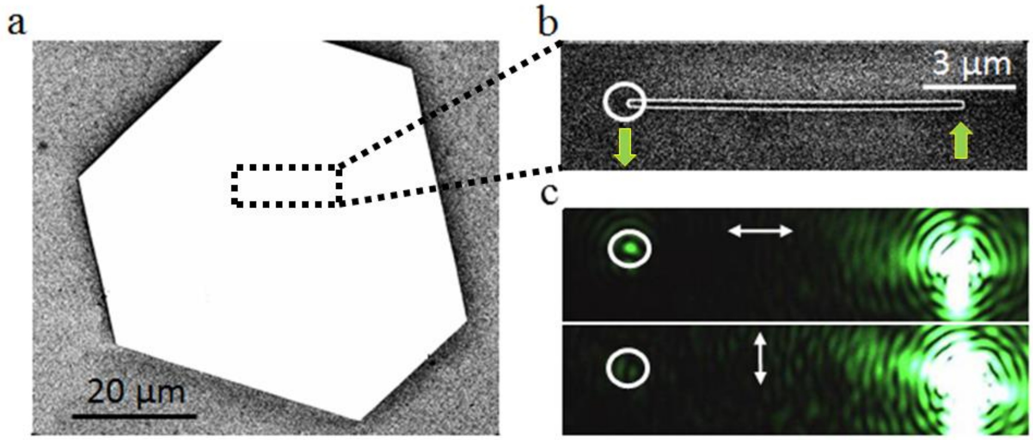

Figure S4 (**a**) SEM image of a Ag crystal flake. (**b**) SEM image of a fabricated waveguide (without grating coupler) on Ag crystalline Ag flake (**c**) Optical characterization of the DLSPPW.

S5. Coupling efficiency of GeV-DLSPPW system

We simulate the reflection and propagation losses of the grating out-coupler which derives the reflection around 8% at 600 nm and propagation losses due to the absorption through the grating around 12% (Fig. S5). This gives a ~80% out-coupling efficiency. We should notify that the simulation is performed based on Palik's data[1] for modeling of Ag plate and provides an overestimation for the propagation losses. For the grating structure that is made on Ag crystal, we estimate 4% losses due to the propagation, and therefore 88% out-coupling efficiency.

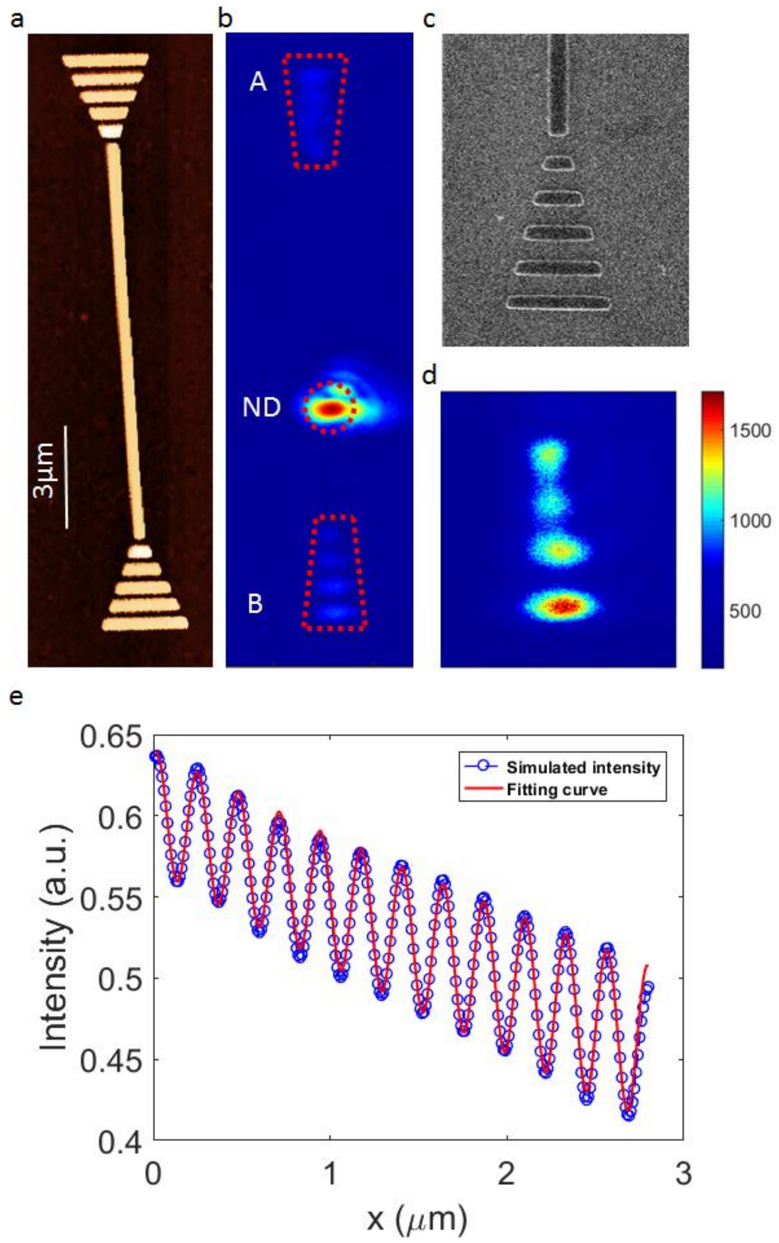

Figure S5 AFM image of the fabricated waveguide on Ag flake (a), and CCD camera image of the whole structure where the nanodiamond is excited and a fluorescence image of the focal plane is taken (b). The 1/e propagation length, L_P, is extracted from the fluorescence signals at the two ends using $P_A/P_B = \exp[(L_A - L_B)/L_P]$, in which $L_A = 8$ μm and $L_B = 4$ μm. We assume symmetric coupling in two directions, uniform losses across the waveguide and the same out-coupling efficiency at the grating ends. The collected data are fitted to obtain the propagation length of 33 ± 3 μm for the GeV-DLSPPW hybrid system on Ag crystal flake that is even higher than the NV- DLSPPW system on Ag film, indicating a low material loss for the single crystalline Ag flakes. (c,d) SEM image of the grating outcoupler and the corresponding CCD image. (e) Simulated intensity I (blue) at the distance of x from the waveguide end to the beginning of the outcoupler). The results are fitted to obtain the reflection loss ($|R|$) around 8% using $I = |E_{in} + E_r|^2$, where $E_{in} = E_0 exp(-ikx)$, and $E_r = E_0 exp(-ikL)R.exp(-ik(L - x))$. L is 2.8 μm, k and E_0 denote the wavenumber, and the incident field, respectively.

S6: Controlled placement of nanodiamonds in plasmonic nanostructures

Positioning of nanodiamonds relative to the coordinates of the markers, and deterministic placement of a waveguide embedding a pre-selected nanodiamond is illustrated in Figure S7. Figure S7(b) and Figure S7(c) show fluorescent image from the nanodiamond and corresponding Gaussian fits for locating x and y position, respectively.

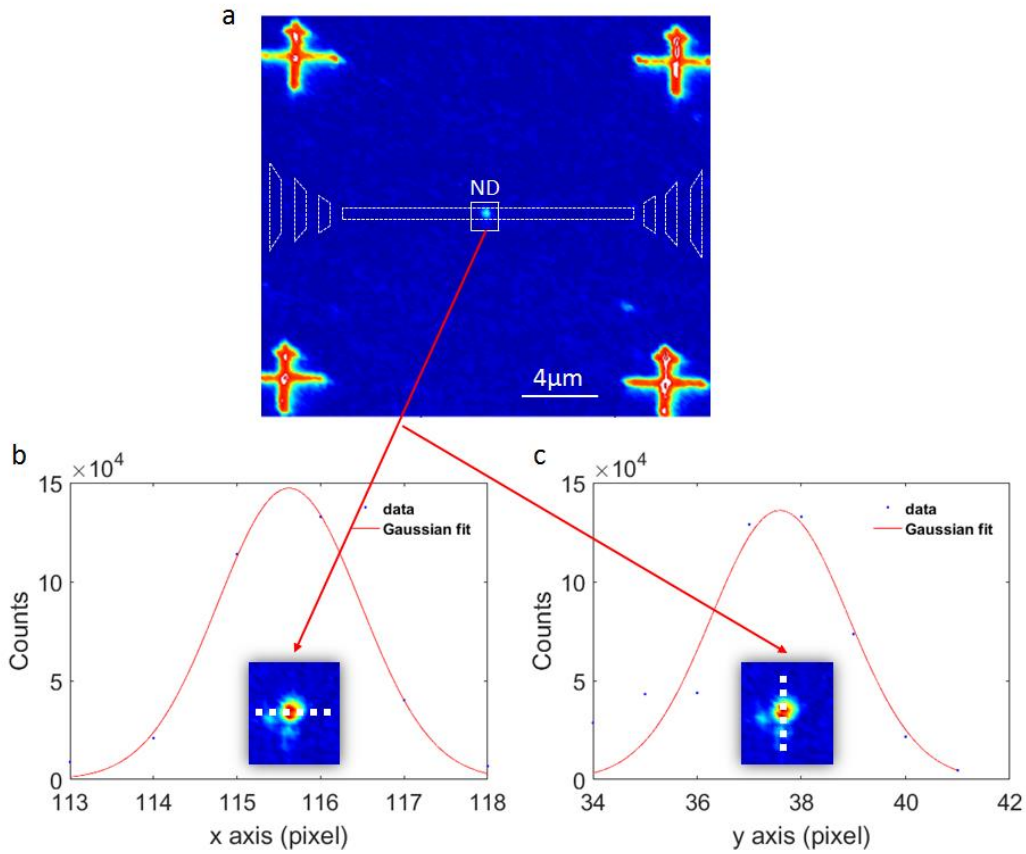

Figure **S6**: Controlled placement of nanodiamonds in plasmonic nanostructures. (**a**) Fluorescence image from a selected region of Ag flake defined by four cross markers. Two spots inside squares indicate nanodiamonds (NDs). White dashed lines indicate location of a waveguide for a pre-determined nanodiamond. (**b**, **c**) Positioning of x and y coordinates for the nanodiamonds using Gaussian fit.

Reference

1. Palik E. *Handbook of Optical Constants of Solids*. Orlando: Academic Press;1985.
